# Psychosocial outcomes of a non-dieting based positive body image community program for overweight adults: a pilot study

**DOI:** 10.1186/2050-2974-1-S1-O55

**Published:** 2013-11-14

**Authors:** Lisa Bloom, Leah Brennan, Beth Shelton, Melissa Bengough

**Affiliations:** 1 School of Health Sciences, RMIT University, Australia; 2 School of Psychology, Australian Catholic University, Australia; 3 Southern Health Wellness Recovery Adult Outpatient Eating Disorder Service, Australia; 4 Monashlink Community Health Service, Australia

## 

The limited success of traditional diet focused weight management interventions has lead to the development of alternative non-dieting approaches. This study evaluated the impact of a community based non-dieting positive body image program for overweight/obese adults.

Participants enrolled in the 8 week 'No More Diets' (NMD) group program completed questionnaires assessing disordered eating thoughts and behaviours, body image, motivation for exercise and psychopathology pre- and post-treatment.

Participants (n=17; 16 female; 19-78 years; BMI 25.2kg/m^2^-55.9kg/m^2^) reported elevated levels of eating disorder psychopathology, body shape pre-occupation, depression, anxiety and stress compared to community norms (*p*<.05) at baseline. Following the treatment there were significant improvements in body shape pre-occupation, shape concern and eating attitudes (p < .05), and moderate to large effect sizes (0.3-0.35) for improvements in reported weight concern, eating competence, stress and health evaluation. There were no changes in reported dietary restraint, emotional eating, uncontrolled eating, or eating concern.

The NMD program was particularly beneficial for improving body image and shape concern but did not improve other disordered eating psychopathology. Addressing these body image factors may help to address some of the perpetuating factors of obesity and disordered eating, which are often not addressed in traditional diet based weight loss interventions.

This abstract was presented in the **Body Image** stream of the 2013 ANZAED Conference.

